# Supplementary material for: Scalable Synthesis of TRPV1 Antagonist Bipyridinyl Benzimidazole Derivative via the Suzuki–Miyaura Reaction and Selective SeO2 Oxidation
Source: Molecules. 2023 Jan 13;28(2):836. doi: 10.3390/molecules28020836 (PMC9860766; doi:10.3390/molecules28020836)

Scalable Synthesis of TRPV1 Antagonist Bipyridinyl Benzimidazole Derivative via Suzuki–Miyaura Reaction and Selective SeO_2 Oxidation

Author List:
Joon-Hwan Lee [1,2], Jiduck Kim [2], Hakwon Kim [1,*]

1 Department of Applied Chemistry, Global Center for Pharmaceutical Ingredient Materials, Kyung Hee University, Yongin 17104, Gyeonggi-do, Korea.
2 Daewoong Pharmaceuticals Co.,Ltd, Yongin 17104, Gyeonggi-do, Korea
* Correspondence: hwkim@khu.ac.kr (H.K.); Tel.: +823-1201-2459 (H.K.)

Supporting Information

Section S1: Characterization of Compounds

Characterization Data for compound 4
^{1}H NMR (500 MHz, MeOD) δ 8.87 (dd, J = 4.9, 1.6 Hz, 1H), 8.51 (d, J = 2.3 Hz, 1H), 8.31 (dd, J = 8.1, 1.6 Hz, 1H), 7.86 (dd, J = 8.1, 2.4 Hz, 1H), 7.67 (dd, J = 8.1, 4.9 Hz, 1H), 7.44 (d, J = 8.0 Hz, 1H), 2.63 (s, 3H).

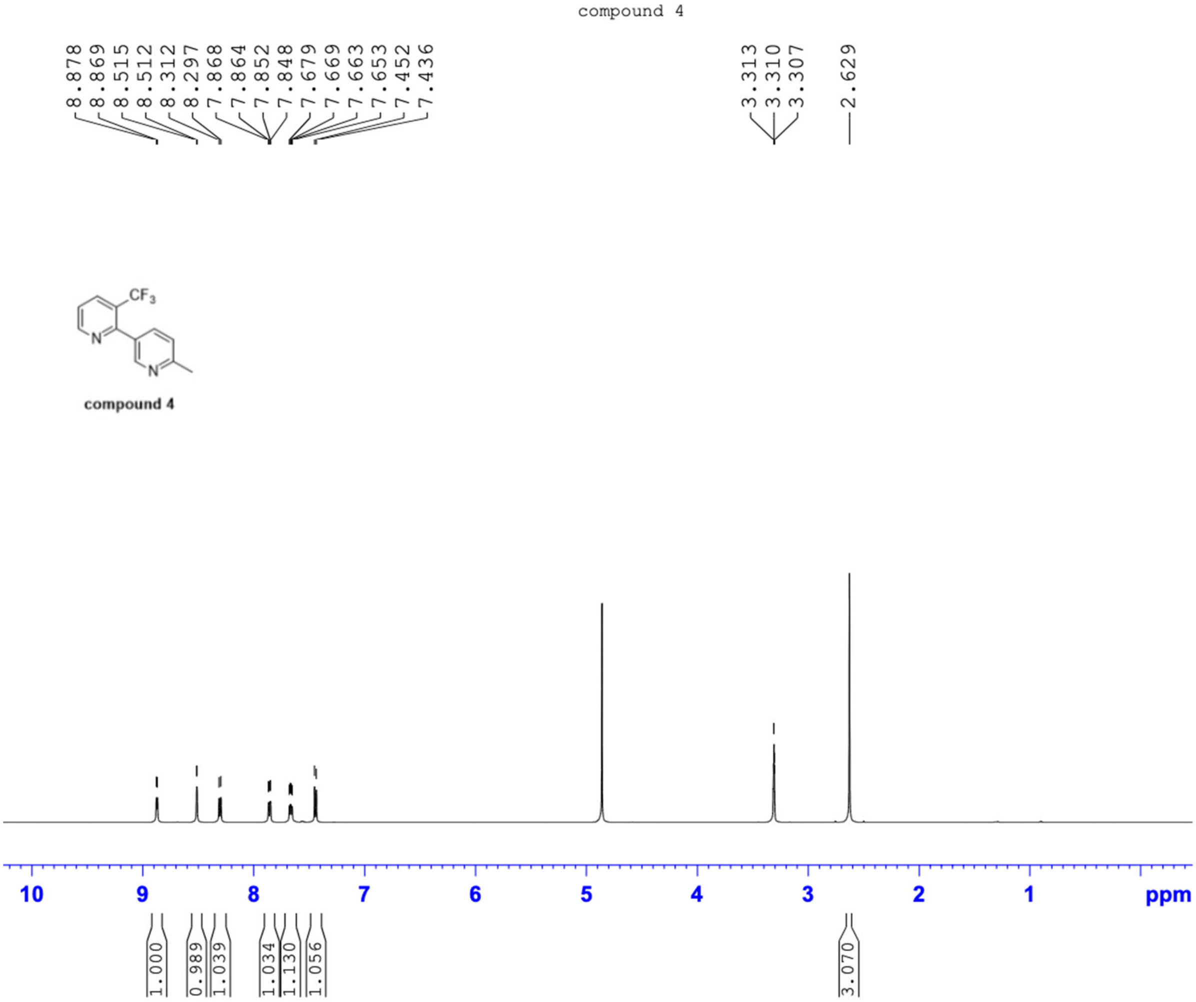

^{13}C NMR (126 MHz, MeOD) δ 160.12, 156.09, 153.46, 148.98, 138.70, 136.75, 134.01, 126.61 (q, J = 31.92 Hz, CCF_3), 124.97 (q, J = 273.13 Hz, CF_3), 124.42, 124.24, 23.74.

compound 4

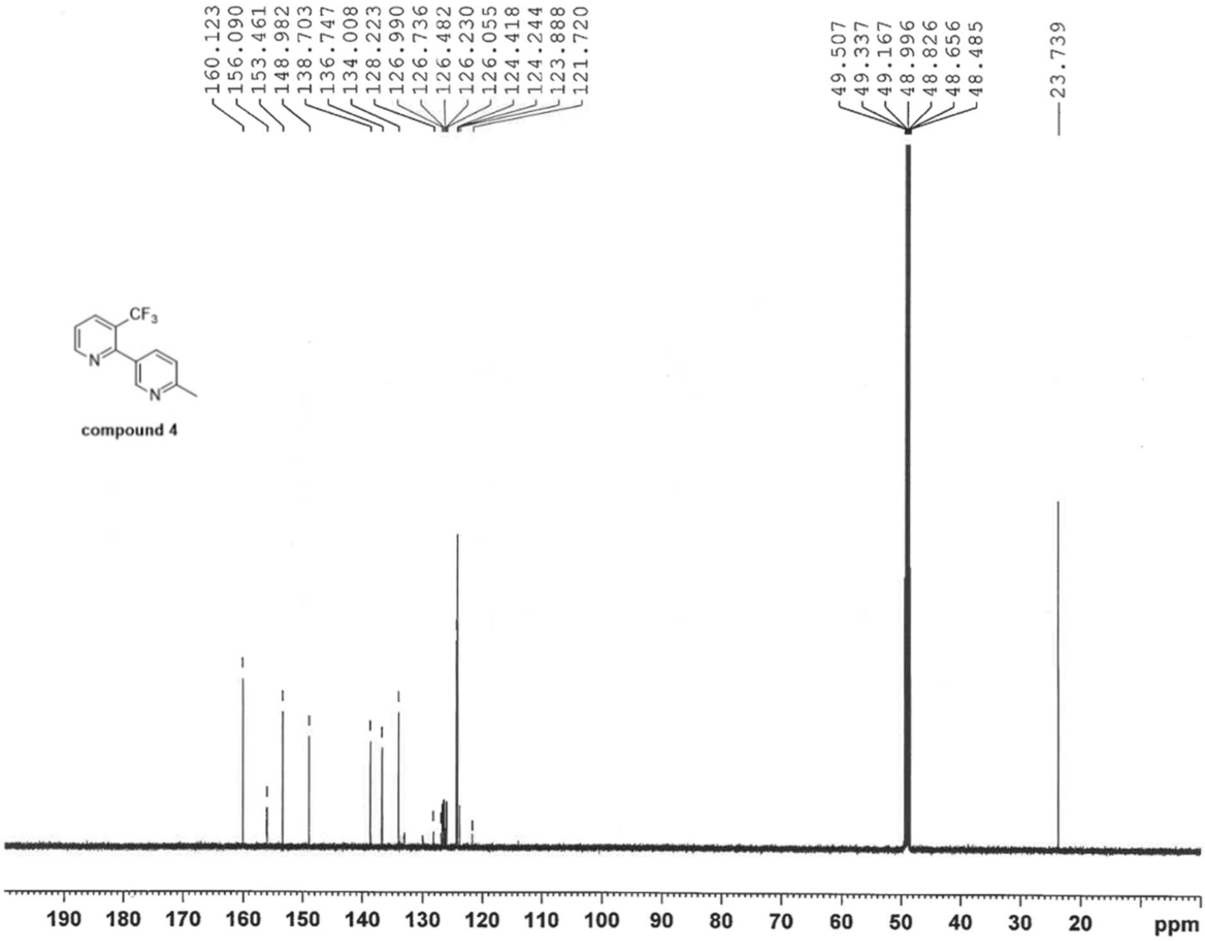
160.123
156.090
153.461
148.982
138.703
136.747
134.008
128.223
126.990
126.736
126.482
126.230
126.055
124.418
124.244
123.888
121.720
49.507
49.337
49.167
48.996
48.826
48.656
48.485
23.739
CF3
N
N
compound 4
190 180 170 160 150 140 130 120 110 100 90 80 70 60 50 40 30 20 ppm

HRMS (ESI) (m/z): calcd for $[C_{12}H_9F_3N_2]^+$ 239.07906 $[M+H]^+$, found 239.07904

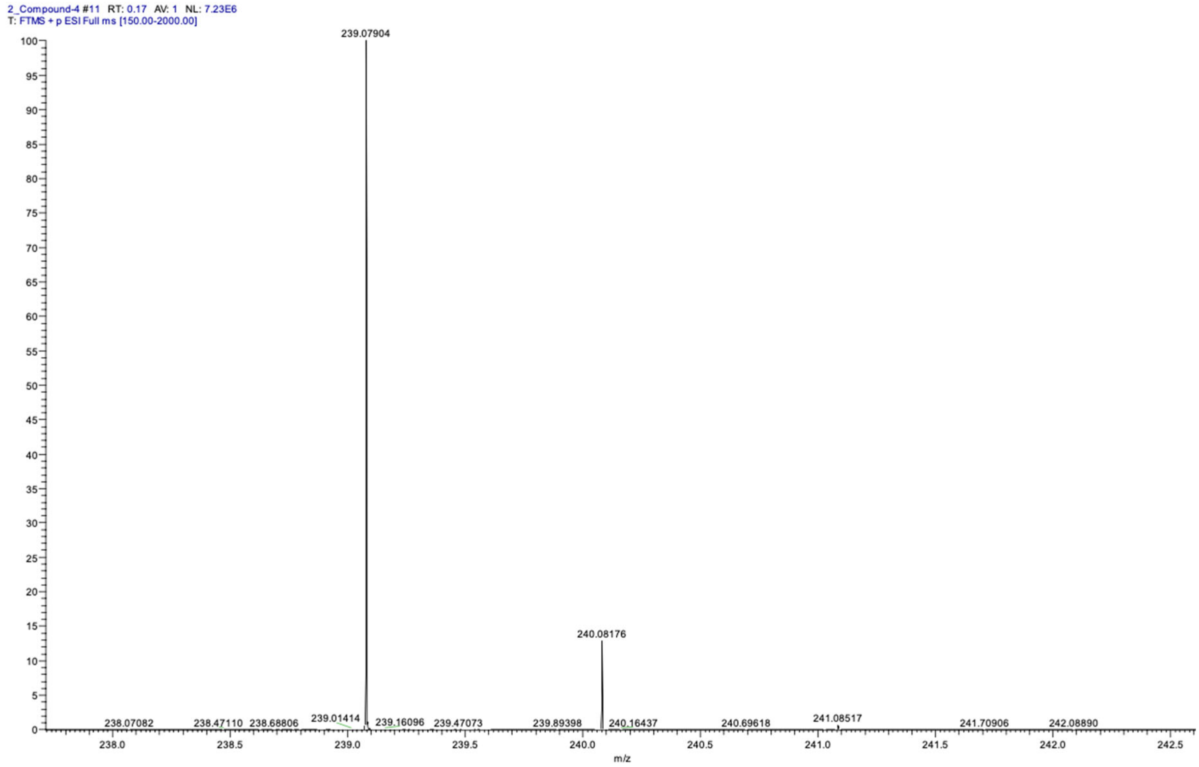

Characterization Data for compound 5

^{1}H NMR (500 MHz, MeOD) δ 8.92 (dd, J = 4.9, 1.6 Hz, 1H), 8.78 (d, J = 2.2 Hz, 1H), 8.34 (dd, J = 8.2, 1.6 Hz, 1H), 8.29 (d, J = 8.1 Hz, 1H), 8.14 (dd, J = 8.1, 2.2 Hz, 1H), 7.71 (dd, J = 8.1, 4.9 Hz, 1H).

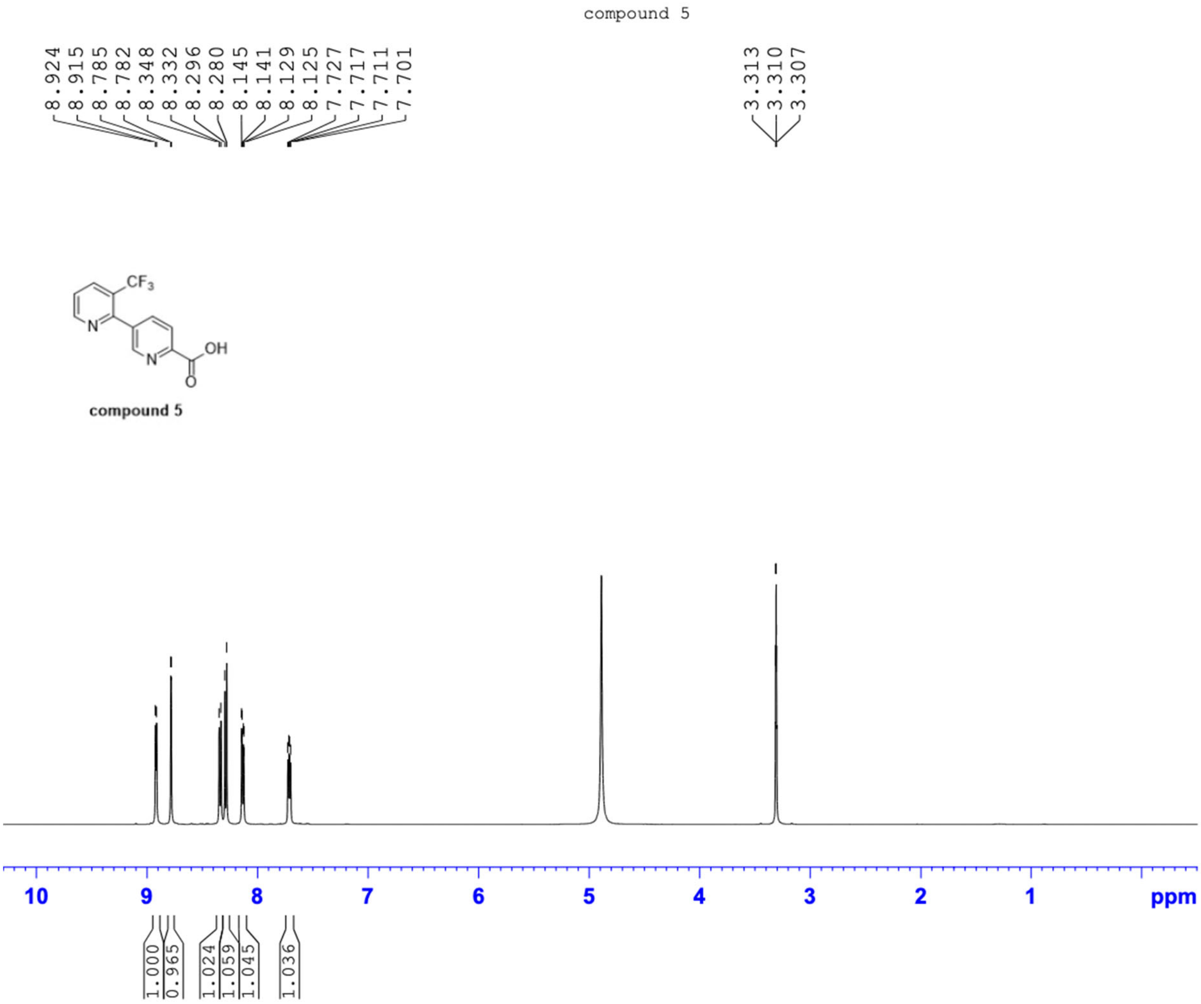

^{13}C NMR (126 MHz, MeOD) δ 167.12, 155.13, 153.66, 149.97, 149.34, 139.58, 139.33, 136.75, 126.67 (q, J = 32.17 Hz, CCF$_3$), 125.94, 124.89, 124.86 (q, J = 273.21 Hz, CF$_3$).

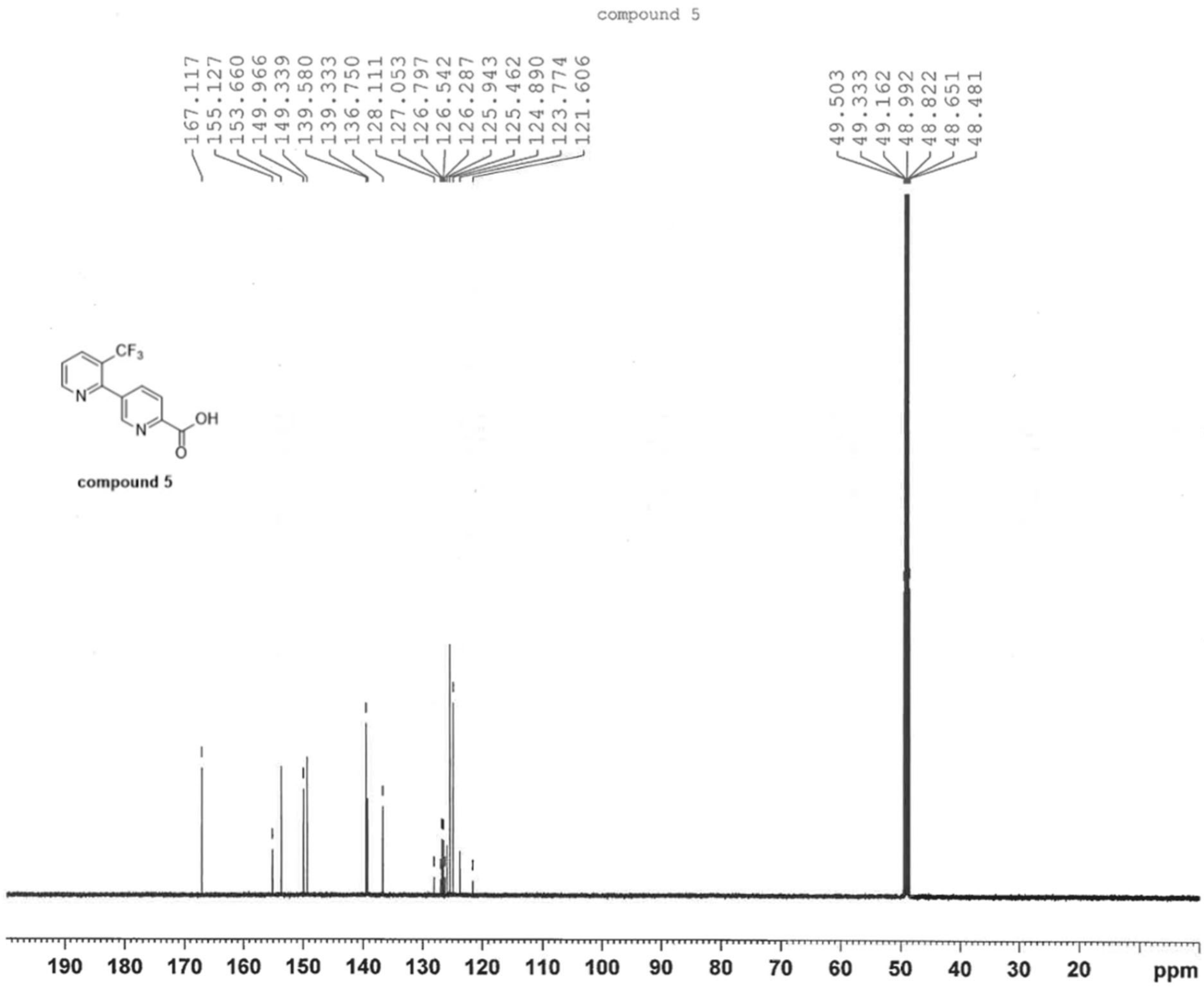

HRMS (ESI) (m/z): calcd for $[C_{12}H_7F_3N_2O_2]^+$ 269.05324 $[M+H]^+$, found 269.05322.

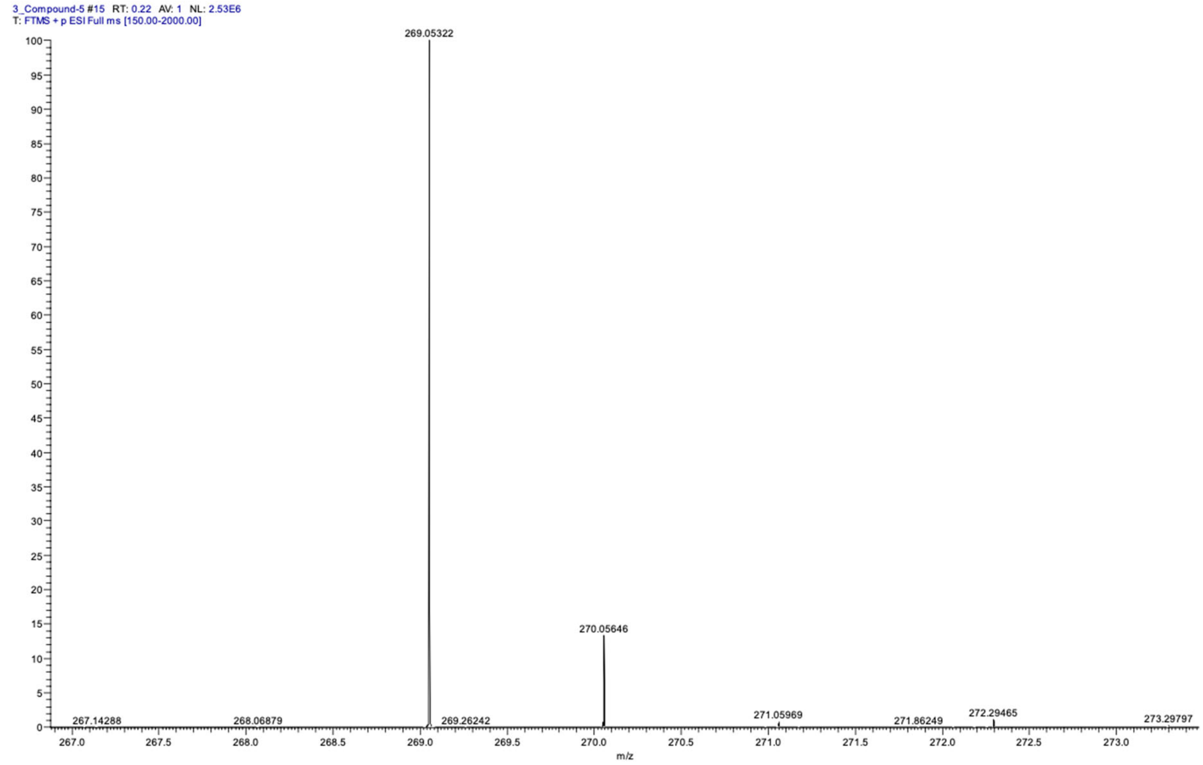

Characterization Data for compound 6

^{1}H NMR (500 MHz, MeOD) δ 8.88 (dd, J = 5.0, 1.6 Hz, 1H), 8.62 (d, J = 2.1 Hz, 1H), 8.31 (dd, J = 8.1, 1.6 Hz, 1H), 7.99 (dd, J = 8.1, 2.2 Hz, 1H), 7.77 (d, J = 8.1 Hz, 1H), 7.67 (dd, J = 8.1, 4.9 Hz, 1H).

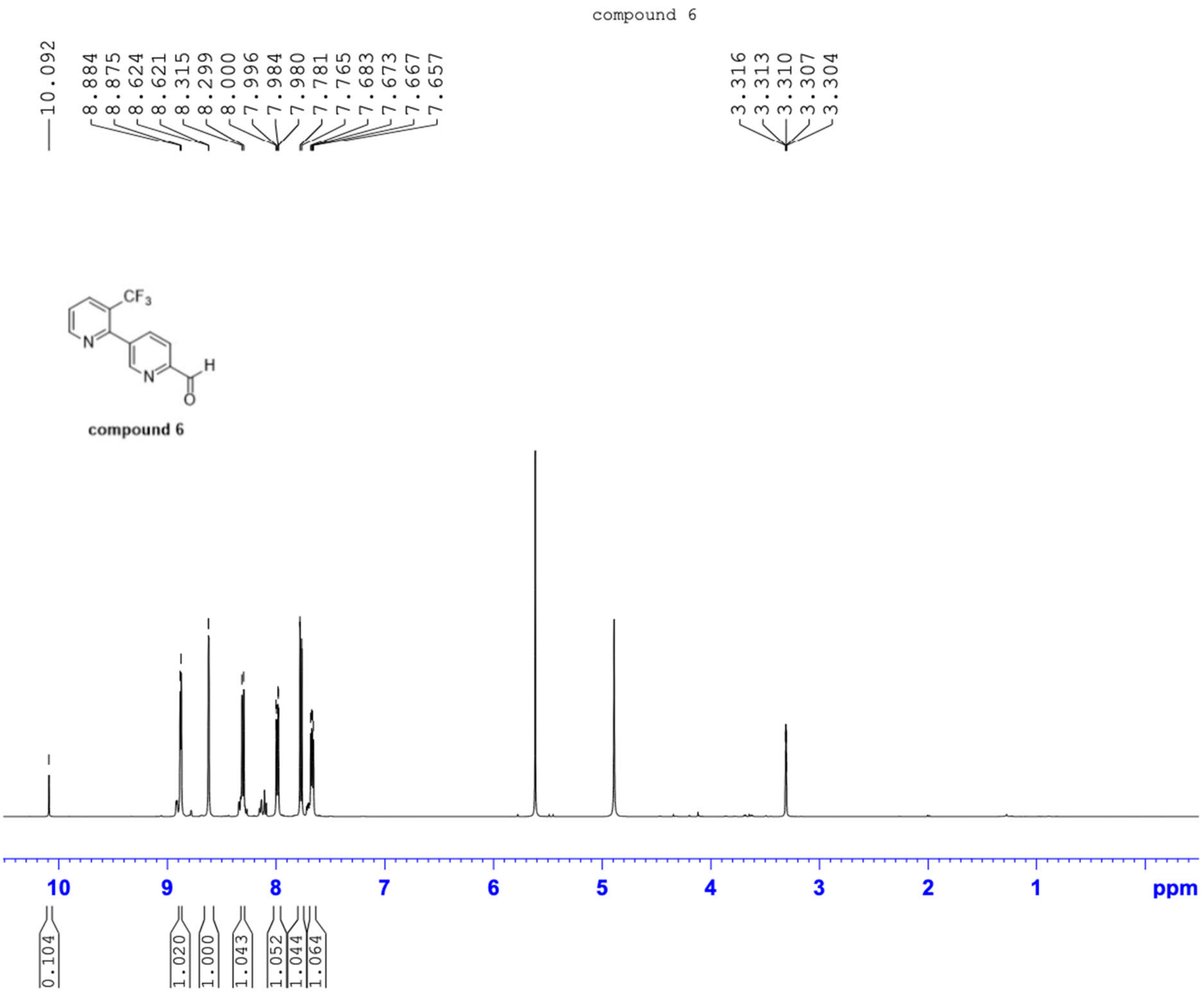

^{13}C NMR (126 MHz, MeOD) δ 193.52, 160.99, 155.78, 153.47, 148.87, 138.85, 136.66, 136.21, 126.51 (q, J = 31.92 Hz, CCF_3), 124.49, 124.88 (q, J = 273.25 Hz, CF_3), 121.24.

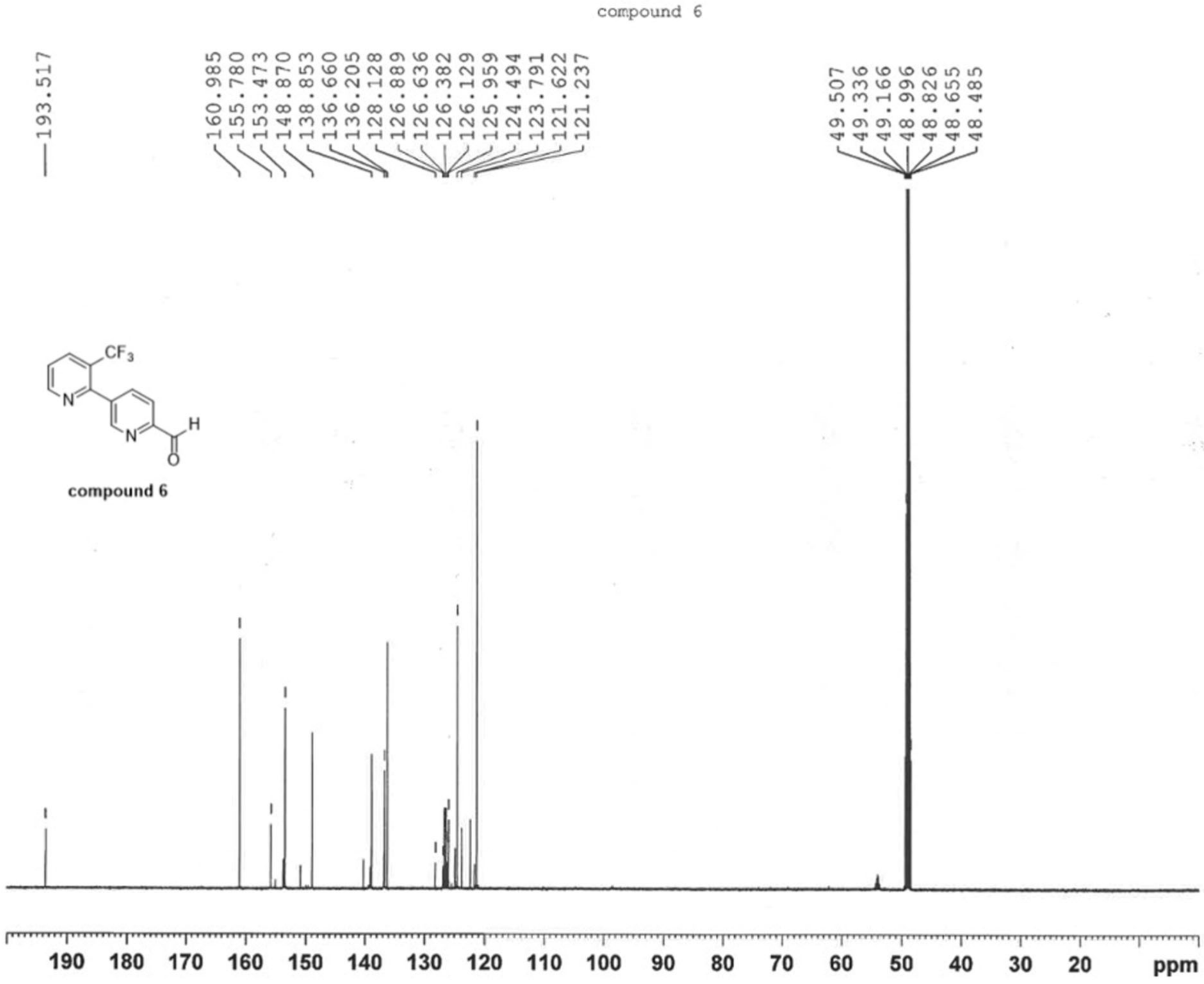

HRMS (ESI) (m/z): calcd for $[C_{12}H_7F_3N_2O]^+$ 253.05832 $[M+H]^+$, found 253.05835.

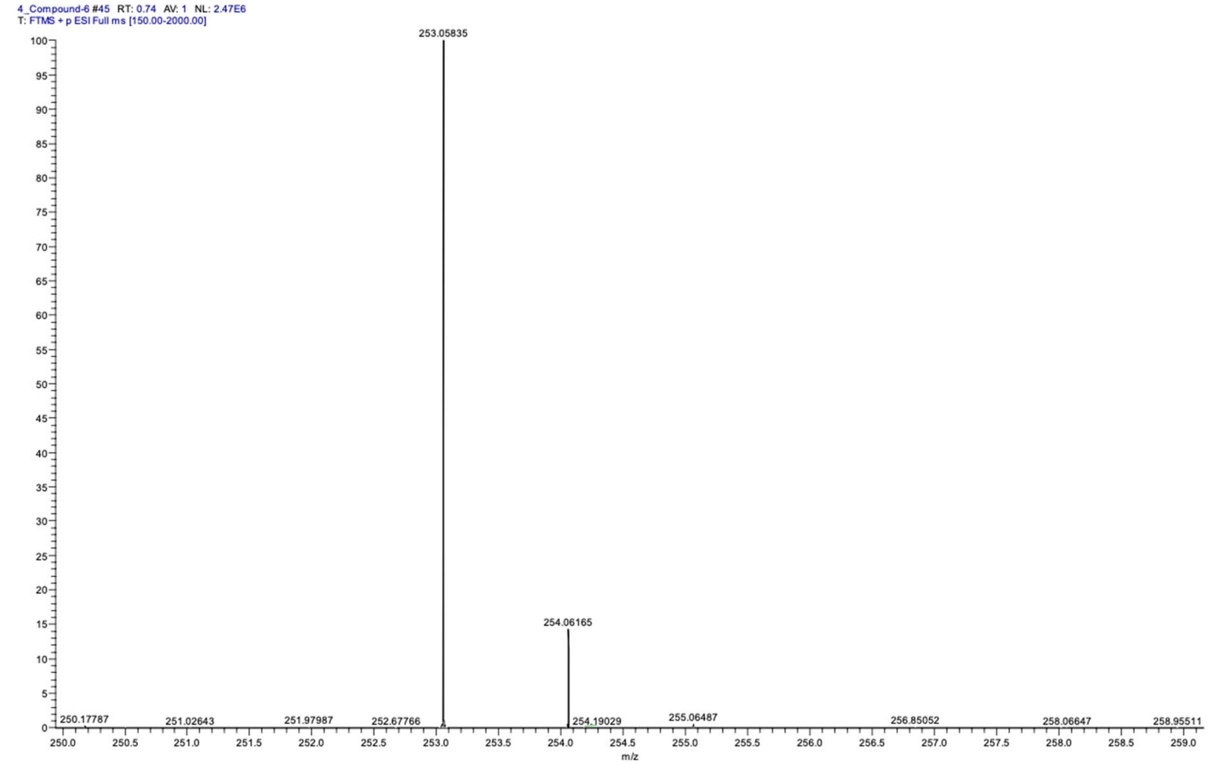

Characterization Data for compound 1

^{1}H NMR (500 MHz, MeOD) δ 8.89 (dd, J = 4.8, 1.6 Hz, 1H), 8.81 (d, J = 2.1 Hz, 1H), 8.36 (d, J = 8.1 Hz, 1H), 8.31 (dd, J = 8.2, 1.6 Hz, 1H), 8.07 (dd, J = 8.1, 2.2 Hz, 1H), 7.79 (s, 1H), 7.67 (dd, J = 8.1, 4.9 Hz, 1H), 7.55 (d, J = 8.1 Hz, 1H), 7.39 (dd, J = 8.5, 1.9 Hz, 1H).

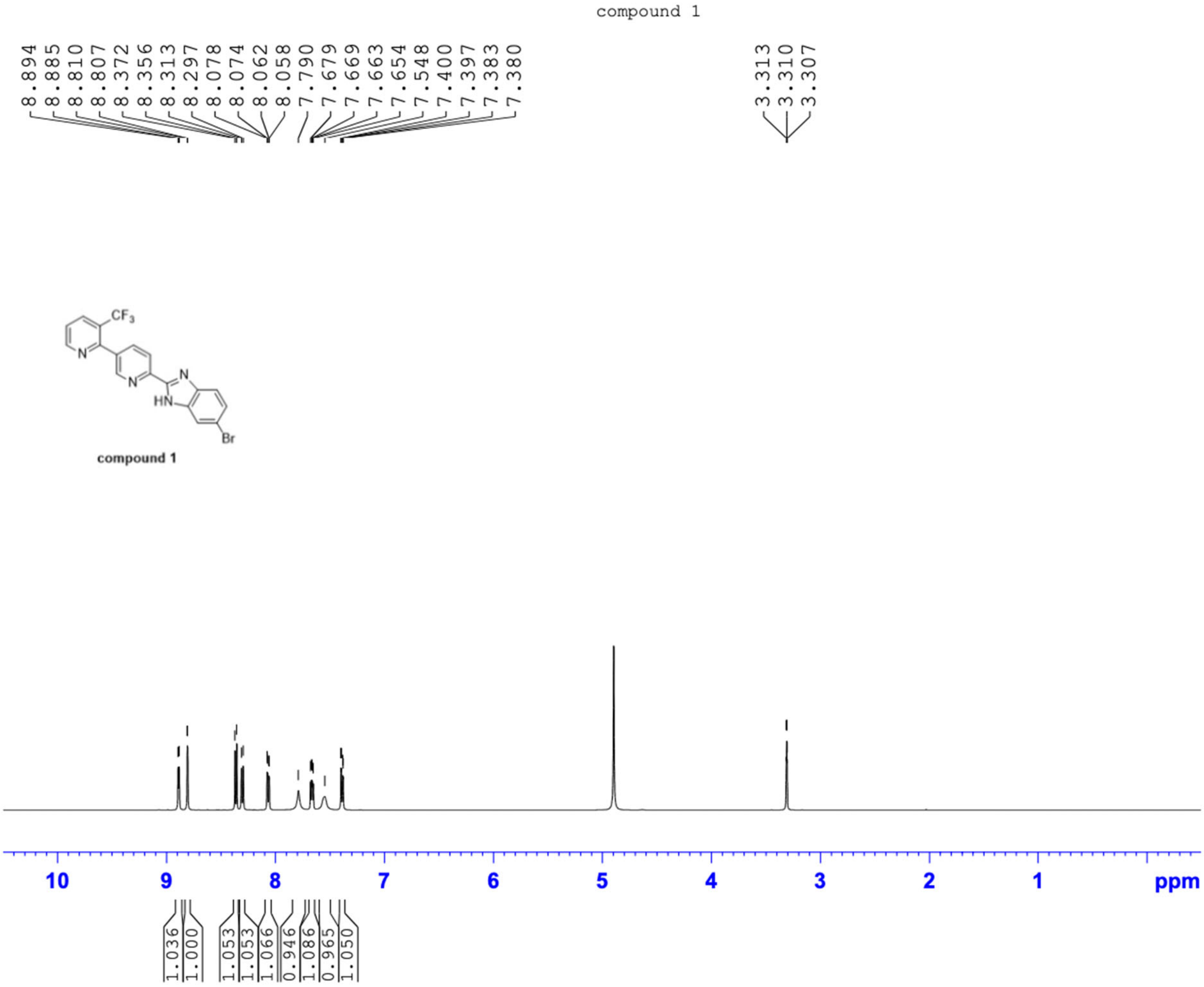

^{13}C NMR (126 MHz, MeOD) δ (ppm) : 153.84, 152.96, 151.32, 148.88, 148.17, 137.74, 135.62, 125.68, 124.01 (q, J = 32.09 Hz, CCF_3), 123.68 (q, J = 273.92 Hz, CF_3), 123.56, 120.92, 115.16.

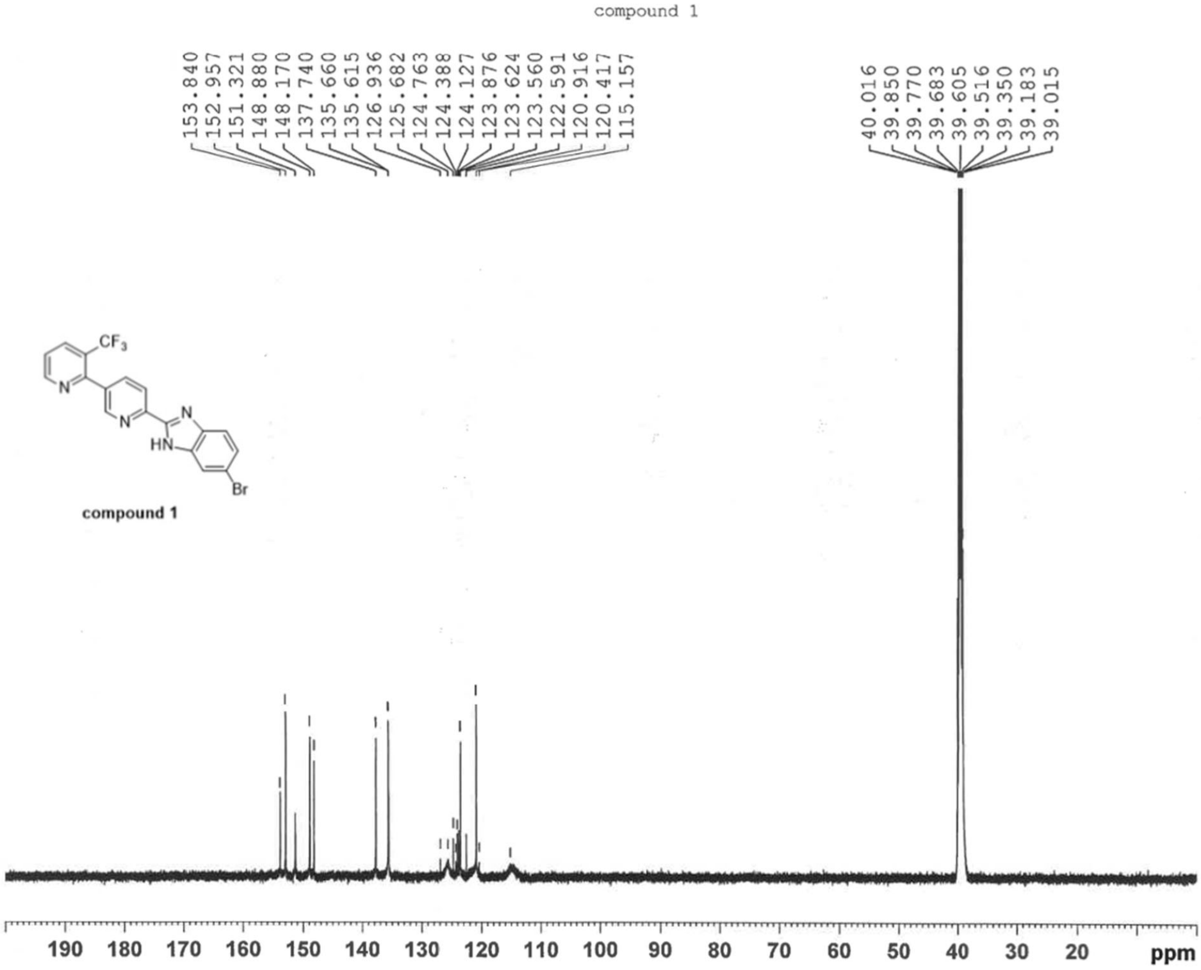

HRMS (ESI) (m/z): calcd for $[C_{18}H_{10}BrF_3N_4]^+$ 419.01137 $[M+H]^+$, found 419.01138.

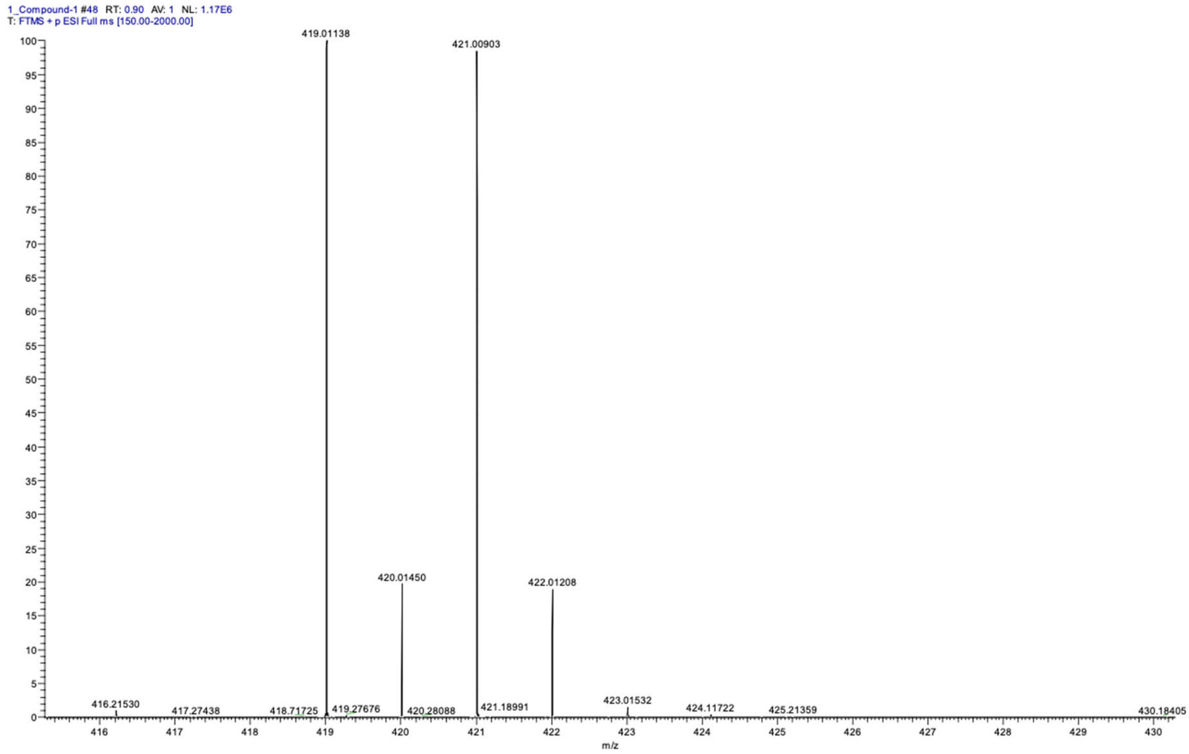

Supplement: Supplementary file 1 [file molecules-28-00836-s001.zip › molecules-2062285-supplementary.pdf]
